# Supplementary material for: MetaRibo-Seq measures translation in microbiomes
Source: Nat Commun. 2020 Jun 29;11:3268. doi: 10.1038/s41467-020-17081-z (PMC7324362; doi:10.1038/s41467-020-17081-z)
Supplement: Supplementary file 10 — Supplementary Data 7 [file 41467_2020_17081_MOESM10_ESM.zip › File2/Confidence_High_Taxonomy/262268_out.krona.html]

Javascript must be enabled to view this page.

members
magnitude
magnitudeUnassigned
count
unassigned
taxon
rank

262268\_out

2

superkingdom
2
2

phylum
201174
1

class
1760
1

85006
1
order

family
1
1268

genus
32207
1

species
1

SRS148193\_contig\_number\_73112
172042

976
1
phylum

class
200643
1

171549
1
order

815
1
family

genus
1
816

species
821

SRS014855\_contig\_number\_2764
1
